# Real-World Survival Comparisons Between Radiotherapy and Surgery for Metachronous Second Primary Lung Cancer and Predictions of Lung Cancer–Specific Outcomes Using Machine Learning: Population-Based Study

**DOI:** 10.2196/53354

**Published:** 2024-06-12

**Authors:** Yue Zheng, Ailin Zhao, Yuqi Yang, Laduona Wang, Yifei Hu, Ren Luo, Yijun Wu

**Affiliations:** 1 Division of Thoracic Tumor Multimodality Treatment Cancer Center, West China Hospital Sichuan University Chengdu China; 2 Laboratory of Clinical Cell Therapy West China Hospital Sichuan University Chengdu China; 3 Department of Hematology West China Hospital Sichuan University Chengdu China; 4 West China School of Medicine Sichuan University Chengdu China

**Keywords:** metachronous second primary lung cancer, radiotherapy, surgical resection, propensity score matching analysis, machine learning

## Abstract

**Background:**

Metachronous second primary lung cancer (MSPLC) is not that rare but is seldom studied.

**Objective:**

We aim to compare real-world survival outcomes between different surgery strategies and radiotherapy for MSPLC.

**Methods:**

This retrospective study analyzed data collected from patients with MSPLC between 1988 and 2012 in the Surveillance, Epidemiology, and End Results (SEER) database. Propensity score matching (PSM) analyses and machine learning were performed to compare variables between patients with MSPLC. Survival curves were plotted using the Kaplan-Meier method and were compared using log-rank tests.

**Results:**

A total of 2451 MSPLC patients were categorized into the following treatment groups: 864 (35.3%) received radiotherapy, 759 (31%) underwent surgery, 89 (3.6%) had surgery plus radiotherapy, and 739 (30.2%) had neither treatment. After PSM, 470 pairs each for radiotherapy and surgery were generated. The surgery group had significantly better survival than the radiotherapy group (*P*<.001) and the untreated group (563 pairs; *P*<.001). Further analysis revealed that both wedge resection (85 pairs; *P*=.004) and lobectomy (71 pairs; *P*=.002) outperformed radiotherapy in overall survival for MSPLC patients. Machine learning models (extreme gradient boosting, random forest classifier, adaptive boosting) demonstrated high predictive performance based on area under the curve (AUC) values. Least absolute shrinkage and selection operator (LASSO) regression analysis identified 9 significant variables impacting cancer-specific survival, emphasizing surgery’s consistent influence across 1 year to 10 years. These variables encompassed age at diagnosis, sex, year of diagnosis, radiotherapy of initial primary lung cancer (IPLC), primary site, histology, surgery, chemotherapy, and radiotherapy of MPSLC. Competing risk analysis highlighted lower mortality for female MPSLC patients (hazard ratio [HR]=0.79, 95% CI 0.71-0.87) and recent IPLC diagnoses (HR=0.79, 95% CI 0.73-0.85), while radiotherapy for IPLC increased mortality (HR=1.31, 95% CI 1.16-1.50). Surgery alone had the lowest cancer-specific mortality (HR=0.83, 95% CI 0.81-0.85), with sublevel resection having the lowest mortality rate among the surgical approaches (HR=0.26, 95% CI 0.21-0.31). The findings provide valuable insights into the factors that influence cumulative cancer-specific mortality.

**Conclusions:**

Surgical resections such as wedge resection and lobectomy confer better survival than radiation therapy for MSPLC, but radiation can be a valid alternative for the treatment of MSPLC.

## Introduction

Lung cancer has become a leading cause of cancer-related deaths worldwide [1]. With the rapid development of screening tools and therapeutic strategies, survival outcomes of lung cancer patients have encouragingly improved, especially for early-stage non-small cell lung cancer (NSCLC), which has a 5-year survival rate as high as 90% [2]. For cancer survivors, longer survival may well lead to a higher probability of developing a second primary cancer. In recent years, metachronous second primary lung cancer (MSPLC) has been commonly observed among survivors with previously treated lung cancer. Thakur et al [3] reported that MSPLC occurred in 2.95% of patients with initial primary lung cancer (IPLC) in the Surveillance, Epidemiology, and End Results (SEER) database. According to the study by Surapaneni et al [4], the risk of developing a second lung cancer is the highest in the first year and continues to be high at 10 years. The surveillance and management of patients with MSPLC have become an urgent issue.

For patients with an initial, early-stage lung cancer, surgical resection remains the most effective treatment. However, there is still a lack of guidelines to assess tumor resectability in patients with MSPLC. Several studies have confirmed the feasibility of surgery for MSPLC [5-9]. Remarkably, patients with MSPLC with previously resected lung cancer may be in poor physical condition and have insufficient lung function reserve, and another surgical procedure may not be appropriate. Thus, an alternative treatment is required for patients with inoperable MSPLC.

Radiation therapy is an effective treatment choice for patients with MSPLC and has fewer complications and impairments. Stereotactic body radiotherapy has recently been reported to have similar survival outcomes as surgery in patients with early-stage lung cancer [10,11]. Previous studies have shown that radiotherapy is a safe and feasible treatment for MSPLC, but whether it can compare with surgery in terms of survival outcomes remains debated [12,13]. Therefore, in this population-based study, the initial step involved conducting propensity score matching (PSM) analyses to compare the survival outcomes of patients who underwent surgical resection with those who received radiotherapy for multiple synchronous primary lung cancers. Furthermore, specific focus was placed on comparing the outcomes of common surgical methods, namely lobectomy and wedge resection, with those of radiotherapy for patients with MSPLC. To enhance the accuracy of the predictions, state-of-the-art machine learning (ML) techniques were used, and multiple algorithms were used to develop robust prediction models.

## Methods

### Data Source

Data for all patients diagnosed with MSPLC included in this retrospective study were sourced from the SEER database [14], covering approximately 30% of cancer patients in the United States. Data pertaining to these patients were extracted from 9 cancer registries and augmented with additional treatment information from regions including Atlanta, Connecticut, Detroit, Hawaii, Iowa, New Mexico, San Francisco–Oakland, Seattle–Puget Sound, and Utah. The data set's most recent follow-up information was updated in November 2018. This study aimed to prognosticate the outcomes for patients with MSPLC. In adherence to the established guidelines for the development and reporting of ML predictive models in biomedical research [15], we meticulously maintained precision and clarity throughout our research process.

### Preparation of Data for Model Building

Patients aged ≥20 years who were diagnosed with MSPLC were identified from the SEER database. We defined MSPLC according to the criteria set by Martini and Melamed [16]. We only included patients with 2 primary lung tumors with a diagnostic interval between the tumors ≥4 years, because it is difficult to distinguish a primary lung tumor from relapse or metastasis when the interval is <4 years [17]. The initial inclusion criteria were as follows: (1) primary sites of the 2 tumors were the lung and bronchus (International Classification of Diseases for Oncology [ICD-O]-3/World Health Organization [WHO] 2008, Third Edition), (2) the time of diagnosis for the IPLC was from January 1988 to December 2012 (to ensure that all enrolled patients had been followed for enough time), and (3) age was ≥20 years. The exclusion criteria included (1) <4 years between the diagnosis of the 2 primary tumors, (2) distant metastasis, (3) histological type of small cell lung cancer for IPLC or MSPLC, and (4) incomplete follow-up information.

We collected the patients’ demographic features and clinical characteristics, such as age at diagnosis, sex, race (White, Black, other [American Indian/Alaska Native, Asian/Pacific Islander], and unknown), location relationship of the 2 primary tumors (ipsilateral and contralateral), diagnostic interval, year of diagnosis, SEER cancer stage (localized and regional), histological type (adenocarcinoma, squamous cell carcinoma, and other NSCLC), grade, surgical procedure, chemotherapy, and radiotherapy (beam radiation). Sublevel resection was regarded as an extent of resection that was less than lobectomy. For patients diagnosed with IPLC after 2004, additional clinical information such as TNM (tumor [T], extent of spread to the lymph nodes [N], and presence of metastasis [M]) stage (6th edition of the American Joint Committee on Cancer TNM system) and tumor size were available.

### Predictive Models

We used 6 classical ML algorithms, namely extreme gradient boosting (XGB), random forest classifier (RFC), adaptive boosting (ADB), K nearest neighbor (KNN), artificial neural network (ANN), and gradient boosting decision tree (GBDT), to forecast long-term cancer-specific survival (CSS). To select the variables for modeling, the least absolute shrinkage and selection operator (LASSO) regression technique was used. An extensive method was used to determine the optimal combination of variables for each algorithm. The performance and predictive capabilities of over a dozen variables were individually assessed using the models, measured using the area under the receiver operating characteristic curves (AUC of ROCs), and decision curve analysis was conducted. The most effective variables were identified, and additional variables were combined iteratively until the best overall results were obtained. The selection of the optimal modeling approach for each algorithm was determined using 5-fold cross-validation. Furthermore, the contribution of each variable was calculated. Additionally, age-adjusted competing risk regression analysis was conducted using the “cmprsk” package in R to examine the cumulative risk of cancer-specific mortality. This comprehensive approach facilitated a thorough evaluation of the risk factors and outcomes associated with cancer-specific mortality in diverse patient populations.

### Statistical Analyses

All statistical analyses were performed using SPSS 27.0 (IBM Corp) and R software version 4.3.1 [18]. SEER*Stat software version 8.4.2 was used to identify the study population from the SEER database. A 2-tailed *P* value <.05 was considered statistically significant. Continuous parameters such as patients’ age and diagnostic interval are expressed as mean (SD) and were compared between the different treatment groups using Mann-Whitney *U* tests. For categorical parameters, proportions were compared using Pearson chi-square tests. To balance the baseline characteristics between the different treatment groups, PSM analyses were used. Survival curves were plotted using the Kaplan-Meier method and compared using log-rank tests.

### Ethical Considerations

The data used in this research were extracted from the publicly accessible, anonymized SEER database. Given the nature of the SEER database, which contains deidentified patient information and is widely used for epidemiological and clinical research purposes, our study fell within the category of research that is exempt from formal ethical approval and consent requirements. This exemption is consistent with established institutional and local policies regarding the use of publicly available, deidentified data for research purposes [19].

## Results

### Demographic Characteristics

According to our inclusion and exclusion criteria, a total of 2451 patients diagnosed with MSPLC were included in this study. All patients’ baseline characteristics are summarized in Table 1. There were 1137 men and 1314 women, with a mean age of 63.5 (SD 9.2) years. White people accounted for 84.1% (2062/2451) of the study population. The mean diagnostic interval between the 2 primary lung tumors was 101.0 (SD 47.6) months. The year of diagnosis of the IPLC ranged from 1988 to 2012. For IPLC, 264 (10.8%) of the 2451 patients did not undergo any surgical procedure, while 2447 underwent surgical resection, including 295 (295/2447, 12%) sublevel resections, 1786 (1786/2447, 72.9%) lobectomies, and 106 (106/2447, 4.3%) pneumonectomies. Additionally, 465 (465/2451, 19%) patients received chemotherapy, and 489 (489/2451, 20%) underwent radiation therapy for IPLC. Based on treatments for MSPLC, patients were divided into the following 4 subgroups: radiotherapy only (864/2451, 35.3%), surgery only (759/2451, 31%), surgery plus radiotherapy (89/2451, 3.6%), and no treatment (739/2451, 30.2%). The median follow-up time after MSPLC diagnosis was 18 (range: 1-273) months. For the entire study population, the 5-year overall survival (OS) was 34.7%.

**Table 1:** Demographic and clinical characteristics of 2451 patients diagnosed with second primary lung cancer.

Characteristic				Results
Age (years), mean (SD)				63.5 (9.2)
**Race, n (%)**				
	White			2062 (84.1)
	Black			240 (9.8)
	Other			149 (6.1)
**Sex, n (%)**				
	Male			1137 (46.4)
	Female			1314 (53.6)
**Relative location, n (%)**				
	Ipsilateral			815 (33.3)
	Contralateral			1636 (66.7)
Diagnostic interval (months), mean (SD)				101.0 (47.6)
**Initial primary lung cancer**				
	**Year of diagnosis, n (%)**			
		1988-1995	763 (31.1)	
		1996-2003	919 (37.5)	
		2004-2012	769 (31.4)	
	**SEER^a^ stage, n (%)**			
		Localized	1538 (62.7)	
		Regional	913(37.3)	
	**Histology, n (%)**			
		ADC^b^	1399 (57.1)	
		SCC^c^	690 (28.2)	
		Other NSCLC^d^	362 (14.8)	
	**Grade, n (%)**			
		Well differentiated	277 (11.3)	
		Moderately differentiated	844 (34.3)	
		Poorly differentiated	792 (32.3)	
		Undifferentiated	115 (4.7)	
		Unknown	423 (17.3)	
	**Surgery, n (%)**			
		No surgery	264 (10.8)	
		Sublevel resection	295 (12)	
		Lobectomy	1786 (72.9)	
		Pneumonectomy	106 (4.3)	
	**Chemotherapy, n (%)**			
		Yes	465 (19)	
		No/unknown	1986 (81)	
	**Radiotherapy, n (%)**			
		Yes	489 (20)	
		No/unknown	1962 (80)	
**Second primary lung cancer**				
	**Surgery, n (%)**			
		No surgery	1603 (65.4)	
		Wedge resection	295 (12)	
		Segmentectomy	61 (2.5)	
		Other/inseparable sublevel resection	87 (3.5)	
		Lobectomy	352 (14.4)	
		Pneumonectomy	53 (2.2)	
	**Chemotherapy, n (%)**			
		Yes	694 (28.3)	
		No/Unknown	1757 (71.7)	
	**Radiotherapy, n (%)**			
		Yes	953 (38.9)	
		No/Unknown	1498 (61.1)	
	**Treatment, n (%)**			
		Only radiotherapy	964 (35.3)	
		Only surgery	759 (31.0)	
		Surgery + radiotherapy	89 (3.6)	
		None	739 (30.2)	

^a^SEER: Surveillance, Epidemiology, and End Results.

^b^ADC: adenocarcinoma.

^c^SCC: squamous cell carcinoma.

^d^NSCLC: non-small cell lung cancer.

### Radiotherapy Versus Surgery

**Figure 1:**
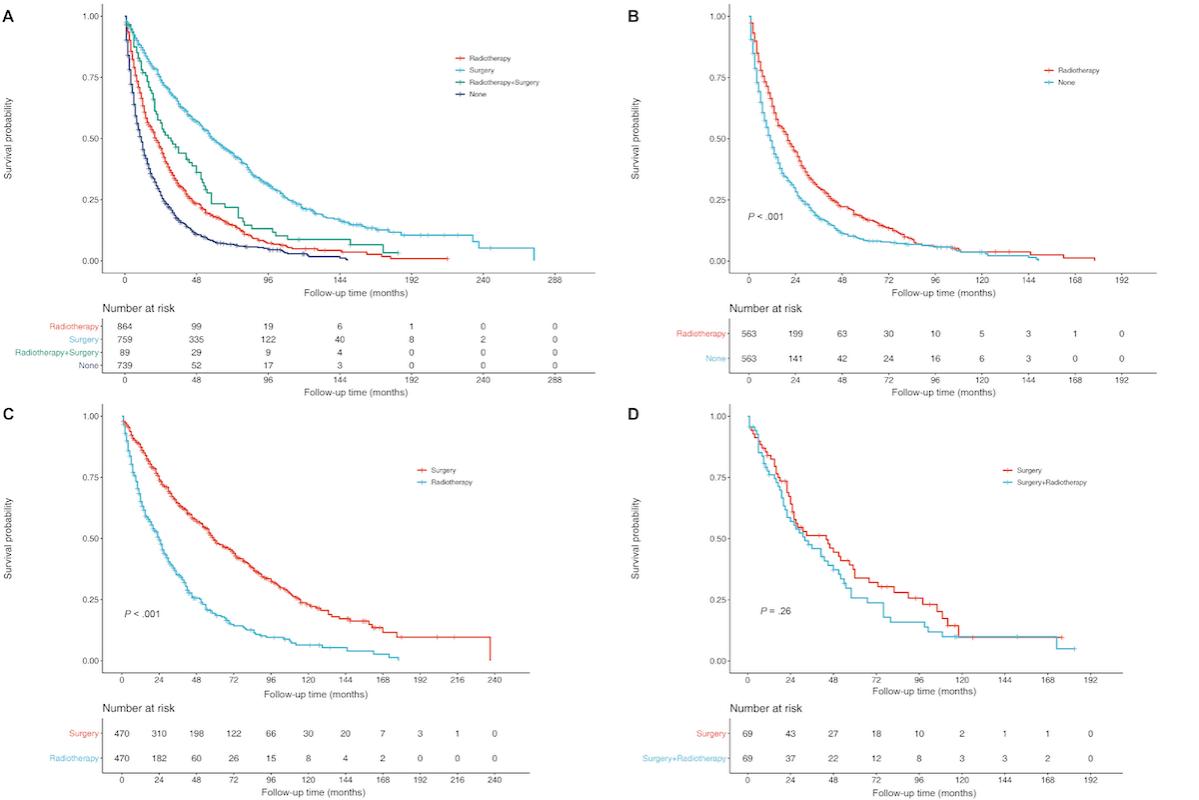
(a) Overall survival of 2451 patients with MSPLC between 1988 and 2012 in different treatment groups before propensity score matching (PSM). (b) Overall survival of radio-therapy and none-treatment after PSM. (c) Overall survival of radiotherapy and surgery after PSM. (d) Overall survival of surgery and surgery plus radiotherapy after PSM.

Before PSM, the distributions of several baseline characteristics were significantly different between the radiotherapy and surgery groups. These included age (*P*<.001); sex (*P*=.005); relative location of the 2 primary tumors (*P*<.001); diagnostic interval (*P*<.001); and IPLC characteristics such as year of diagnosis (*P*=.004), histology (*P*<.001), surgical procedure (*P*<.001), radiotherapy (*P*=.04), and chemotherapy for MSPLC (*P*<.001; Table 2). Figure 1A shows the survival outcomes among the 4 treatment groups (*P*<.001). Patients who only received radiotherapy had worse survival than those who underwent surgical resection but better survival than the no treatment group.

To evaluate the role of radiotherapy in terms of treatment for MSPLC, multiple PSM analyses were performed to compare radiotherapy with no treatment, surgery, and surgery plus radiotherapy. After PSM (ratio: 1:1; caliper=0.01), all baseline characteristics were matched well between the corresponding comparison groups (Table 2 and Tables S1 and S2 in Multimedia Appendix 1). As shown in Figure 1, the radiotherapy group had significantly better survival outcomes than the no treatment group (*P*<.001; Figure 1B) but significantly worse survival outcomes than the surgery group (*P*<.001; Figure 1C). However, radiotherapy seemed to not improve the survival outcome among patients who received surgery for MSPLC (*P*=.26; Figure 1D).

**Table 2:** Comparison of baseline characteristics between surgery and radiotherapy for second primary lung cancer before and after propensity score matching (PSM).

Characteristic					Before PSM										After PSM						
					Radiation (n=864)			Surgery (n=759)			*P* value			Radiation (n=470)				Surgery (n=470)			*P* value
Age (years), mean (SD)					63.9 (8.9)			62.1 (9.0)			<.001			63.0 (8.8)				62.7 (9.1)			.55
**Race, n (%)**											.10										.75
	White			737 (85.3)			642 (84.6)						401 (85.3)				393 (83.6)				
	Black			85 (9.8)			63 (8.3)						39 (8.3)				45 (9.6)				
	Other			42 (4.9)			54 (7.1)						30 (6.4)				32 (6.8)				
**Sex, n (%)**											.005										.95
	Male			417 (48.3)			313 (41.2)						201 (42.8)				203 (43.2)				
	Female			447 (51.7)			446 (58.8)						269 (57.2)				267 (56.8)				
**Relative location, n (%)**											<.001										.73
	Ipsilateral			321 (37.2)			208 (27.4)						152 (32.3)				146 (31.1)				
	Contralateral			543 (62.8)			551 (72.6)						318 (67.7)				324 (68.9)				
Diagnostic interval (months), mean (SD)					104.4 (48.7)			95.8 (45.3)			<.001			99.1 (43.5)				100.9 (50.5)			.56
**IPLC^a^**																					
	**Year of diagnosis**									.004										.93	
		1988-1995	242 (28)			256 (33.7)						135 (28.7)				135 (28.7)					
		1996-2003	313 (36.2)			286 (37.7)						174 (37)				169 (36)					
		2004-2012	309 (35.8)			217 (28.6)						161 (34.3)				166 (35.3)					
	**SEER^b^ stage**									.499										.73	
		Localized	560 (64.8)			505 (66.5)						300 (63.8)				306 (65.1)					
		Regional	304 (35.2)			254 (33.5)						170 (36.2)				164 (34.9)					
	**Histology**									<.001										.82	
		ADC^c^	451 (52.2)			495 (65.2)						275 (58.5)				275 (58.5)					
		SCC^d^	280 (32.4)			173 (22.8)						125 (26.6)				131 (27.9)					
		Other NSCLC^e^	133 (15.4)			91 (12)						70 (14.9)				64 (13.6)					
	**Grade**									.06										≥.99	
		Well differentiated	82 (9.5)			106 (14)						50 (10.6)				52 (11.1)					
		Moderately differentiated	295 (34.1)			259 (34.1)						167 (35.5)				168 (35.7)					
		Poorly differentiated	295 (34.1)			231 (30.4)						149 (31.7)				148 (31.5)					
		Undifferentiated	42 (4.9)			33 (4.3)						22 (4.7)				23 (4.9)					
		Unknown	150 (17.4)			130 (17.1)						82 (17.4)				79 (16.8)					
	**Surgery**									<.001										.98	
		No surgery	102 (11.8)			47 (6.2)						45 (9.6)				42 (8.9)					
		Sublevel resection	100 (11.6)			105 (13.8)						59 (12.6)				61 (13)					
		Lobectomy	616 (71.3)			591 (77.9)						353 (75.1)				355 (75.5)					
		Pneumonectomy	46 (5.3)			16 (2.1)						13 (2.8)				12 (2.6)					
	**Chemotherapy**									.77										.87	
		Yes	155 (17.9)			131 (17.3)						91 (19.4)				88 (18.7)					
		No/unknown	709 (82.1)			628 (82.7)						379 (80.6)				382 (81.3)					
	**Radiotherapy**									.04										.93	
		Yes	176 (20.4)			123 (16.2)						86 (18.3)				88 (18.7)					
		No/unknown	688 (79.6)			636 (83.8)						384 (81.7)				382 (81.3)					
**SPLC^f^**																					
	**Chemotherapy**									<.001										≥.99	
		Yes	318 (36.8)			91 (12)						88 (18.7)				87 (18.5)					
		No/unknown	546 (63.2)			668 (88)						382 (81.3)				383 (81.5)					

^a^IPLC: initial primary lung cancer.

^b^SEER: Surveillance, Epidemiology, and End Results.

^c^ADC: adenocarcinoma.

^d^SCC: squamous cell carcinoma.

^e^NSCLC: non-small cell lung cancer.

^f^SPLC: second primary lung cancer.

### Radiotherapy Versus Wedge Resection or Lobectomy

**Figure 2:**
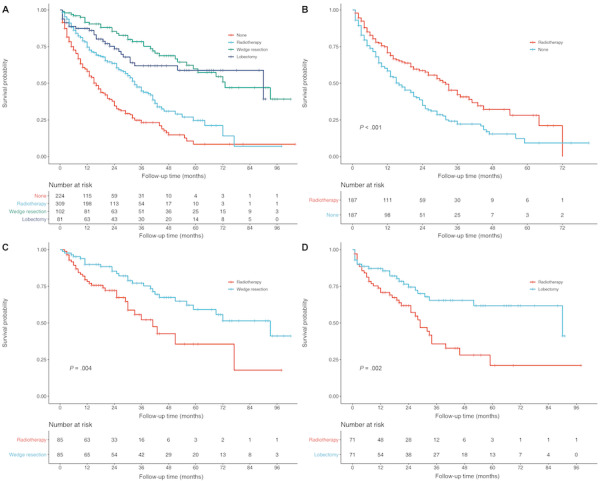
Overall survival of (A) 716 patients with metachronous second primary lung cancer (MSPLC) after 2004 in different treatment groups before propensity score matching (PSM); (B) patients who received radiotherapy or no treatment, after PSM; (C) patients who received radiotherapy or underwent wedge resection, after PSM; (D) patients who received radiotherapy or underwent lobectomy, after PSM.

To further compare survival between radiotherapy and specific surgical procedures, patients with MSPLC diagnosed with IPLC after 2004 were selected. Those who underwent unknown or indefinite sublevel resection, segmentectomy (very few patients) and pneumonectomy for MSPLC were excluded. There were 716 patients included for further analyses. The demographic characteristics are described in Table 3. Before PSM, Figure 2A shows that patients who underwent wedge resection or lobectomy had significantly better OS than those who received radiotherapy, and all of them had significantly better OS than the no treatment group. More clinical parameters such as T and N stage for IPLC and tumor size for MSPLC were matched by PSM, and all parameters were matched well (Tables S3-S5 in Multimedia Appendix 1). Similarly, after PSM, both wedge resection (*P*=.004; Figure 2C) and lobectomy (*P*=.002; Figure 2D) had significantly better OS than radiotherapy. Furthermore, radiotherapy also had greater survival benefits than no treatment (*P*<.001; Figure 2B).

**Table 3:** Demographic and clinical characteristics of 716 patients diagnosed with second primary lung cancer after 2004.

Characteristic				Results
Age (years), mean (SD)				65.8 (9.0)
**Race, n (%)**				
	White		608 (84.9)	
	Black		65 (9.1)	
	Other		43 (6)	
**Sex, n (%)**				
	Male		310 (43.3)	
	Female		406 (56.7)	
**Relative location, n (%)**				
	Ipsilateral		279 (39)	
	Contralateral		437 (61)	
**Interval, mean (SD)**				74.2 (21.4)
**Initial primary lung cancer**				
	**T stage, n (%)**			
		T1	315 (44)	
		T2	277 (38.7)	
		T3	35 (4.9)	
		T4	66 (9.2)	
		Unknown	23 (3.2)	
	**N stage, n (%)**			
		N0	528 (73.7)	
		N1	80 (11.2)	
		N2	97 (13.5)	
		Unknown	11 (1.5)	
	**Histology, n (%)**			
		ADC^a^	406 (56.7)	
		SCC^b^	206 (28.8)	
		Other NSCLC^c^	104 (14.5)	
	**Grade, n (%)**			
		Well differentiated	94 (13.1)	
		Moderately differentiated	275 (38.4)	
		Poorly differentiated	211 (29.5)	
		Undifferentiated	18 (2.5)	
		Unknown	118 (16.5)	
	**Surgery, n (%)**			
		No surgery	131 (18.3)	
		Sublevel resection	106 (14.8)	
		Lobectomy	455 (63.5)	
		Pneumonectomy	24 (3.4)	
	**Chemotherapy, n (%)**			
		Yes	237 (33.1)	
		No/unknown	479 (66.9)	
	**Radiotherapy, n (%)**			
		Yes	174 (24.3)	
		No/unknown	542 (75.7)	
**Second primary lung cancer**				
	**Size (cm), n (%)**			
		0-3	385 (53.8)	
		3-5	71 (9.9)	
		>5	56 (7.8)	
		Unknown	204 (28.5)	
	**Surgery, n (%)**			
		No surgery	533 (74.4)	
		Wedge resection	102 (14.2)	
		Lobectomy	81 (11.3)	
	**Chemotherapy, n (%)**			
		Yes	201 (28.1)	
		No/unknown	515 (71.9)	
	**Radiotherapy, n (%)**			
		Yes	309 (43.2)	
		No/unknown	407 (56.8)	
	**Treatment, n (%)**			
		None	224 (31.3)	
		Only radiation	309 (43.2)	
		Only wedge	102 (14.2)	
		Only lobectomy	81(11.3)	

^a^ADC: adenocarcinoma.

^b^SCC: squamous cell carcinoma.

^c^NSCLC: non-small cell lung cancer.

### ML-Based Cancer-Specific Death Risk Prediction

**Figure 3:**
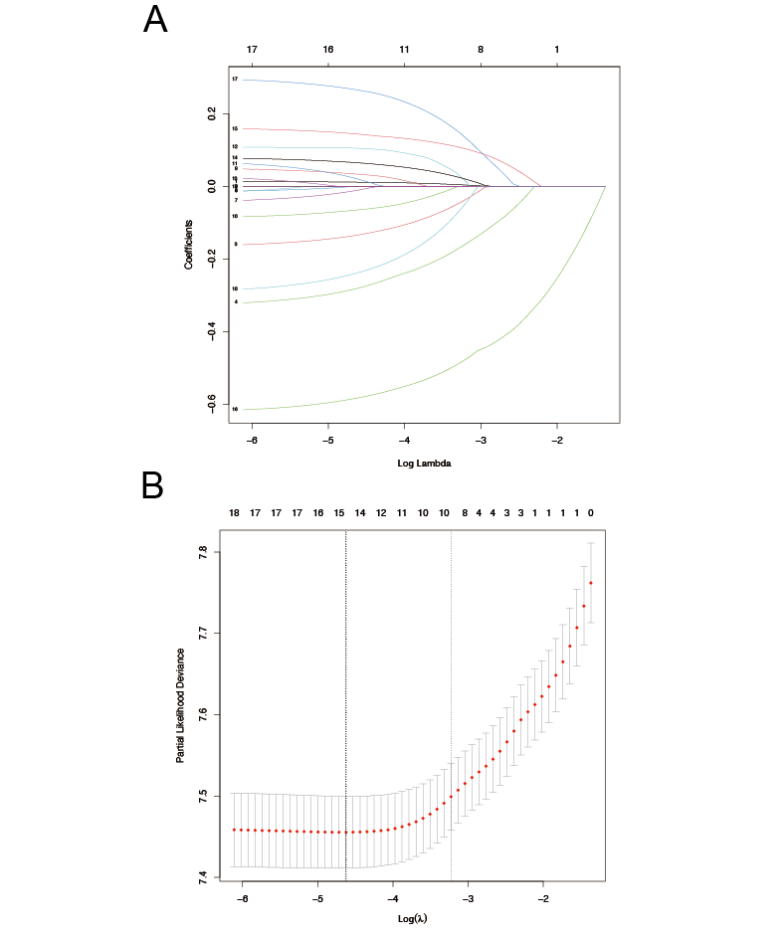
Machine learning model using least absolute shrinkage and selection operator (LASSO) regression analysis for risk prediction of cumulative cancer-specific mortality in patients with metachronous second primary lung cancer (MSPLC): (A) 5-fold cross-validation results and (B) model regression coefficient profile.

**Figure 4:**
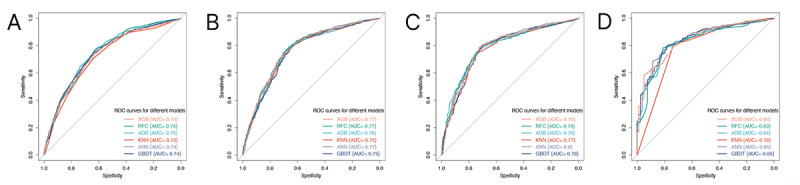
Receiver operating characteristic (ROC) curves for machine learning models for risk prediction of cumulative cancer-specific mortality in patients with metachronous second primary lung cancer (MSPLC): (A) 1-year lymphoma-specific mortality; (B) 3-year lymphoma-specific mortality; (C) 5-year lymphoma-specific mortality; (D) 10-year lymphoma-specific mortality. ADB: adaptive boosting; ANN: artificial neural network; AUC: area under the curve; GBDT: gradient boosting decision tree; KNN: K nearest neighbor; RFC: random forest classifier; ROC: receiver operating characteristic; XGB: extreme gradient boosting.

**Figure 5:**
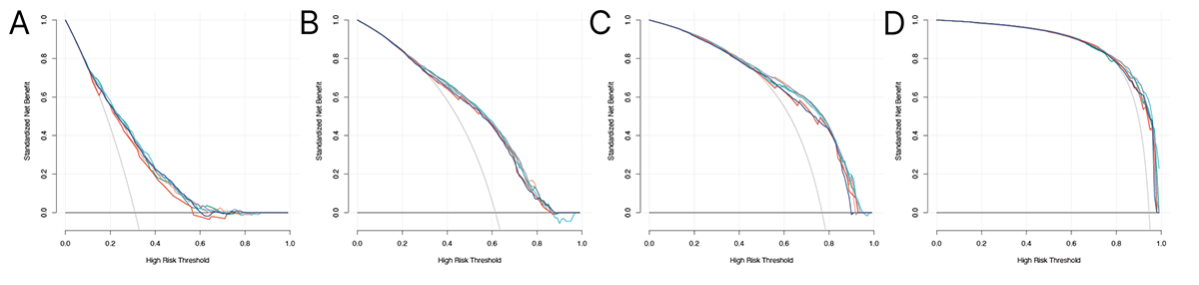
Decision curve analysis for 6 classical machine learning–based models for risk prediction of cumulative cancer-specific mortality in patients with metachronous second primary lung cancer (MSPLC): (A) 1-year lymphoma-specific mortality; (B) 3-year lymphoma-specific mortality; (C) 5-year lymphoma-specific mortality; (D) 10-year lymphoma-specific mortality.

**Figure 6:**
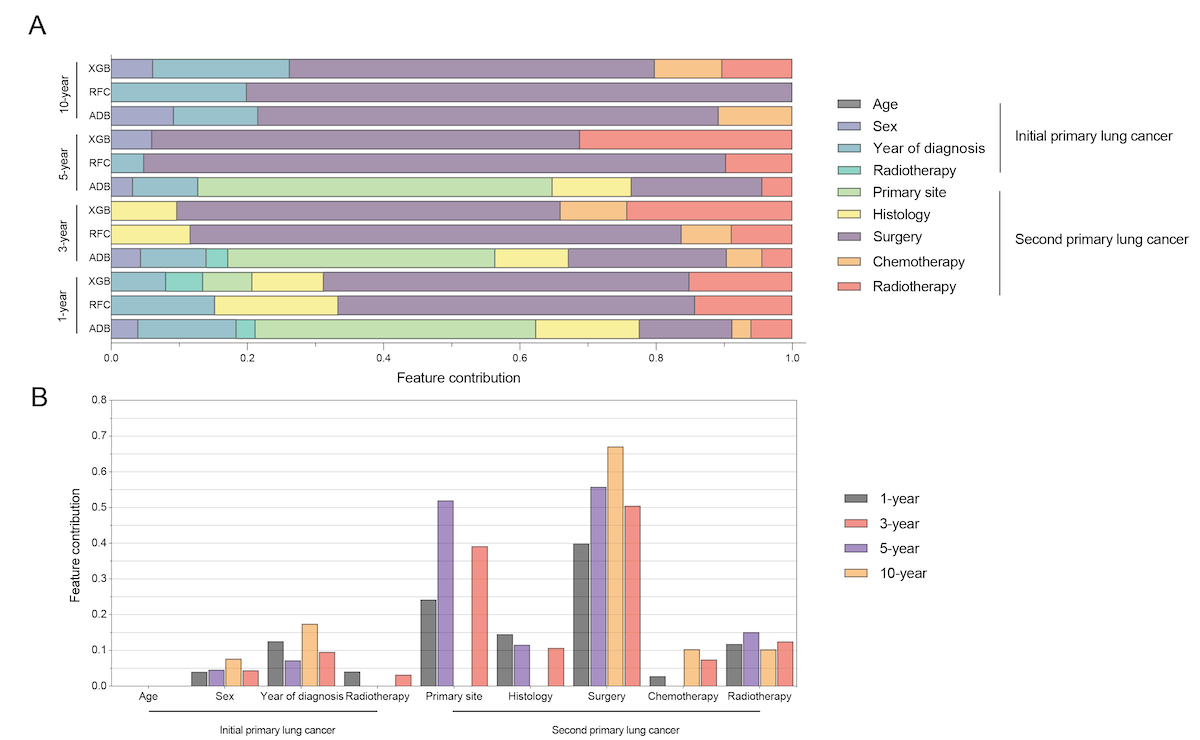
Machine learning model for risk prediction of cumulative cancer-specific mortality in patients with metachronous second primary lung cancer (MSPLC) showing the feature contribution (A) to survival and (B) by cancer characteristics. ADB: adaptive boosting; RFC: random forest classifier; XGB: extreme gradient boosting.

Using LASSO regression, we identified 9 variables that made significant contributions to CSS (Figure 3). These variables encompassed age at diagnosis, sex, year of diagnosis, radiotherapy of IPLC, primary site, histology, surgery, chemotherapy, and radiotherapy of MPSLC. The ML models displayed outstanding performance, as indicated by high AUC values, highlighting the superiority of artificial intelligence in prognostic prediction (Figure 4). The decision curve analyses are depicted in Figure 5. Additionally, we assessed the sensitivity and specificity of each ML model using the maximal Youden index, which represents an optimal balance between true positives and true negatives (Table 4). Through 5-fold cross-validation, the XGB, RFC, and ADB models demonstrated superior performance. In order to gain deeper insights into the relationships between demographic characteristics and long-term outcomes for MSPLC patients, we used these ML algorithms to develop predictive models to assess the 1-year, 3-year, 5-year, and 10-year risks of cumulative cancer-specific mortality based on the aforementioned variables. Consequently, we calculated the contribution of each variable. Notably, we identified the variables associated with CSS at different time intervals (Figure 6). Surgery for MPSLC predominantly and substantially influenced 1-year, 3-year, 5-year, and 10-year CSS. Radiotherapy for MPSLC also had an impact on 1-year, 3-year, 5-year, and 10-year survival, but its effect was comparatively less than that of surgery. The primary site and histology of MPSLC affected 1-year, 3-year, and 5-year CSS, but it had no impact on 10-year CSS. Additionally, radiotherapy for IPLC had an impact on 1-year and 3-year CSS but had minimal influence on 5-year and 10-year survival.

**Table 4:** Performance of machine learning models for risk prediction of long-term cancer-specific survival of patients with second primary lung cancer after 2004.

Model			Sensitivity, %		Specificity, %		AUC^a^ (95% CI)
**1-year cancer-specific survival**							
	XGB^b^	77		60.2		0.73 (0.71-0.75)	
	RFC^c^	76.7		63		0.74 (0.72-0.76)	
	ADB^d^	83.1		54.4		0.75 (0.73-0.77)	
	KNN^e^	70.9		63.6		0.72 (0.70-0.74)	
	ANN^f^	88.2		41.9		0.74 (0.72-0.76)	
	GBDT^g^	90.6		36		0.74 (0.72-0.76)	
**3-year cancer-specific survival**							
	XGB	69.9		73.8		0.77 (0.75-0.79)	
	RFC	75.6		69.2		0.77 (0.75-0.79)	
	ADB	79.3		66.4		0.76 (0.74-0.78)	
	KNN	79.6		64		0.75 (0.73-0.77)	
	ANN	83.6		59.9		0.77 (0.75-0.79)	
	GBDT	84.4		57.6		0.75 (0.73-0.77)	
**5-year cancer-specific survival**							
	XGB	79.6		71.3		0.78 (0.75-0.81)	
	RFC	79.2		71.5		0.79 (0.76-0.82)	
	ADB	75.3		74.7		0.79 (0.76-0.82)	
	KNN	74.3		73.9		0.77 (0.74-0.80)	
	ANN	79.3		71.5		0.80 (0.77-0.83)	
	GBDT	80.1		69.5		0.78 (0.75-0.81)	
**10-year cancer-specific survival**							
	XGB	78.8		74.7		0.84 (0.80-0.88)	
	RFC	78.3		40.7		0.83 (0.79-0.87)	
	ADB	78.4		81		0.84 (0.80-0.88)	
	KNN	80.7		73.4		0.78 (0.72-0.84)	
	ANN	68.8		88.6		0.85 (0.81-0.89)	
	GBDT	79.7		78.5		0.85 (0.81-0.89)	

^a^AUC: area under the curve.

^b^XGB: extreme gradient boosting.

^c^RFC: random forest classifier.

^d^ADB: adaptive boosting.

^e^KNN: K nearest neighbor.

^f^ANN: artificial neural network.

^g^GBDT: gradient boosting decision tree.

### Age-Adjusted Competing Risk Analysis

**Figure 7:**
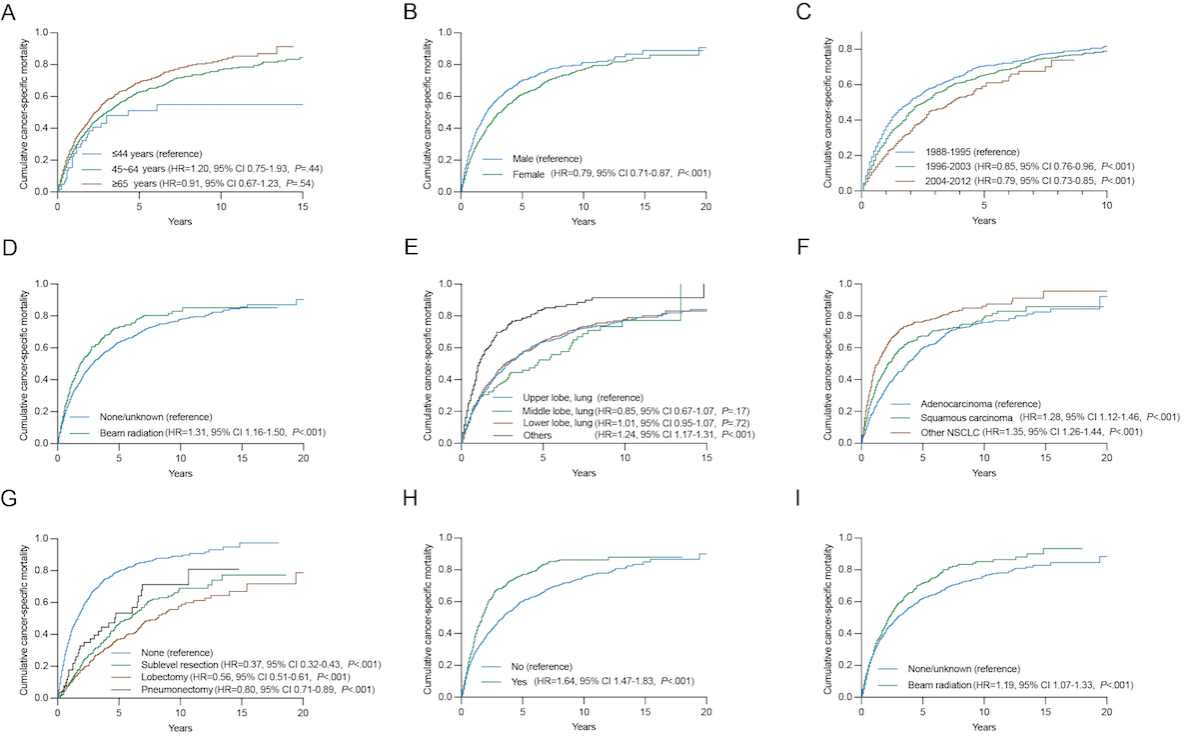
Cumulative cancer-specific mortality per age-adjusted competing risk analysis in subgroup analysis by (A) age, (B) sex, (C) year of diagnosis of the initial primary lung cancer, (D) radiotherapy of the initial primary lung cancer, (E) primary site of the second primary lung cancer, (F) histology of the second primary lung cancer, (G) surgery for the second primary lung cancer, (H) chemotherapy of the second primary lung cancer, (I) radiotherapy of the second primary lung cancer. HR: hazard ratio.

To gain further insights into the cumulative incidence associated with each variable, we conducted competing risk analyses (Figure 7). Female MPSLC patients had lower cumulative cancer-specific mortality (hazard ratio [HR]=0.79, 95% CI 0.71-0.87; *P*<.001). Patients diagnosed with IPLC in more recent years also had lower cumulative cancer-specific mortality: 1996-2003 (HR=0.85, 95% CI 0.76-0.96; *P*<.001); 2004-2012 (HR=0.79, 95% CI 0.73-0.85; *P*<.001). However, patients who received radiotherapy for their IPLC had increased mortality (HR=1.31, 95% CI 1.16-1.50; *P*<.001). The histology of the second primary lung cancer played a significant role, with higher mortality rates for squamous carcinoma than adenocarcinoma (HR=1.28, 95% CI 1.12-1.46; *P*<.001). Moreover, the use of surgery for the second primary lung cancer was associated with lower mortality rates. This was particularly true for sublevel resection (HR=0.37, 95% CI 0.32-0.43; *P*<.001), lobectomy (HR=0.56, 95% CI 0.51-0.61; *P*<.001), and pneumonectomy (HR=0.80, 95% CI 0.71-0.89; *P*<.001). Conversely, the use of chemotherapy or radiotherapy for the second primary lung cancer was associated with increased mortality rates, potentially due to the severity of the patients' initial condition (chemotherapy: HR=1.64, 95% CI 1.47-1.83; *P*<.001; radiotherapy: HR=1.19, 95% CI 1.07-1.33; *P*<.001).

**Figure 8:**
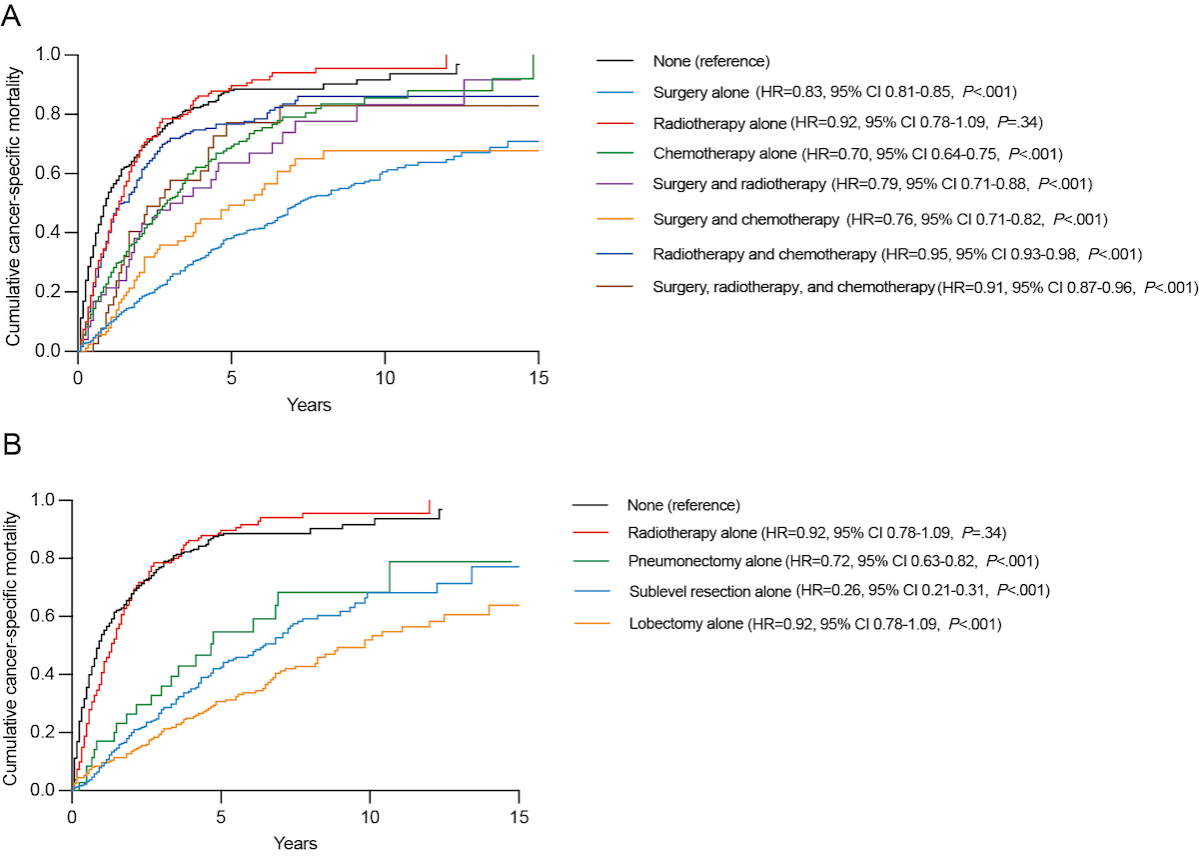
Age-adjusted competing risk analysis to estimate the cumulative cancer-specific mortality with different treatment modalities: (A) treatment for second primary lung cancer, (B) radiotherapy alone versus surgery alone. HR: hazard ratio.

Therefore, we performed additional analyses for the different treatment modalities, as shown in Figure 8. Surgery alone (HR=0.83, 95% CI 0.81-0.85; *P*<.001) had the lowest cancer-specific mortality, followed by surgery and chemotherapy (HR=0.76, 95% CI 0.71-0.82, *P*<.001) and surgery and radiotherapy (HR=0.79, 95% CI 0.71-0.88, *P*<.001). Among different surgical approaches, sublevel resection alone (HR=0.26, 95% CI 0.21-0.31, *P*<.001) had the lowest mortality rate, followed by pneumonectomy alone (HR=0.72, 95% CI 0.63-0.82, *P*<.001) and lobectomy alone (HR=0.92, 95% CI 0.78-1.09, *P*<.001).

## Discussion

### Principal Findings

With the rapid advancement and wide application of low-dose computed tomography for screening of pulmonary nodules, more patients are being diagnosed with MSPLC. Multiple primary lung cancer (MPLC) is a special kind of lung carcinoma that can be categorized into synchronous MPLC and metachronous MPLC. Among these, MSPLC is the most common form of MPLC that can be expected to receive curable management. However, limited progress has been made so far on accurate diagnoses, optimal medical interventions, and prognostic outcomes. In this study, our findings suggest that surgical resections, including wedge resection and lobectomy, contribute to better survival rates than radiation therapy in the context of MSPLC. However, it is important to note that radiation therapy remains a viable and valid alternative for the treatment of MSPLC.

Surgical resection is reportedly feasible for MSPLC and could significantly improve the prognosis [5-9], but the role of radiation therapy in the treatment of MSPLC remains unclear. Considering that patients who previously underwent surgery for IPLC may not tolerate another pulmonary resection, finding optimal alternative treatments is important. Therefore, using the population-based SEER database, this study used PSM analyses and ML techniques to first compare survival outcomes between patients who received radiotherapy or underwent surgical resection for MSPLC.

Of all enrolled patients, most (2187/2451, 89.2%) had undergone surgery for IPLC before (Table 1). However, 65.4% (1603/2451) of the patients with MSPLC did not undergo surgical resection for MSPLC, and 35.3% (864/2451) of them received radiation therapy (Table 1). It could be inferred that a considerable proportion of patients with MSPLC could not tolerate another surgical resection, and radiotherapy might be the predominant alternative treatment for them. Although surgical resection was first recommended for patients with MSPLC, radiation therapy is also important, especially for inoperable cancers. Given that very few studies have focused on long-term survival outcomes after radiotherapy versus surgery, this study may provide a more solid indication in terms of the use of radiotherapy for patients with MSPLC.

Previous studies reported that 5-year OS rates for patients with MSPLC varied, ranging from 26% to 38% [20-22]; these rates are similar to that of our study (34.7% for the entire cohort). The 5-year survival rates were 18.0% for radiotherapy, 49.3% for surgery, 38.8% for surgery plus radiotherapy, and 7.7% for no treatment. Ono et al [13] reported on 19 patients who were diagnosed with MSPLC after lung resection for IPLC and underwent proton beam therapy. Their research showed a 3-year survival rate of 63.2% and a 3-year local control rate of 84.2%, which indicated the safety and feasibility of proton beam therapy for patients with MSPLC. Miyazaki et al [23] compared survival outcomes among metachronous MPLC patients after stereotactic body radiotherapy (N=26) and surgery (N=51) and found no significant differences. The study by Taioli et al [24] included 494 cases from the SEER database and showed that OS was better with surgery than with radiation therapy after the treatment of MSPLC [24]. However, their inclusion criteria were not rigorous enough; the diagnostic interval between the 2 primary lung tumors was too short (6 months), which could fail to exclude patients with relapse or metastasis. Additionally, the analyses were not adjusted for confounding factors, and this might have caused significant bias. In our study, multiple PSM analyses were performed to control for confounding effects. The surgery group had significantly better survival than the radiotherapy group (*P*<.001), and the radiotherapy group had greater survival than the no treatment group (*P*<.001; Figure 1). Therefore, surgical resection should be considered first for patients with MSPLC if their physical condition and pulmonary function reserve permit. For those with an inoperable cancer or who are not willing to undergo another surgery, radiation therapy may be an alternative. Additionally, after PSM, there was no significant difference between the surgery and surgery plus radiotherapy groups (*P*=.26), which indicated that preoperative or postoperative radiotherapy might not increase survival benefits for patients with MSPLC.

Lobectomy remains the commonly accepted standard treatment for resectable NSCLC. In recent years, sublobar resections have been widely reported to be adequate in early-stage NSCLC, resulting in less impairment and greater respiratory function reserve [1,25,26]. However, the prognostic role of sublobar resection among patients with MSPLC has not been clearly clarified. Yang et al [8] identified 454 matched pairs of patients with MSPLC receiving lobectomy or sublobar resection from the SEER database and found that the lobectomy group had significantly better survival than the sublobar resection group. Lee et al [27] concluded that MSPLC had similar survival outcomes with wedge resection and lobectomy by analyzing 625 patients with a diagnostic interval ≥6 months. There have been few studies that have focused on survival outcomes after radiotherapy compared with wedge resection or lobectomy. Thus, to further verify the rigorousness of our study, patients diagnosed after 2004 and with definite therapeutic information (only including no treatment, radiotherapy, wedge resection, and lobectomy) were selected to compare survival outcomes between those undergoing radiotherapy or specific surgical resections. Very few cases underwent segmentectomy (n=16) or surgery plus radiotherapy (n=11) for MSPLC and were excluded. Additionally, T and N stage (American Joint Committee on Cancer, 6th edition) for IPLC and tumor size were also adjusted using PSM analyses. Of the 716 patients diagnosed after 2004, 53.8% had MSPLC with a tumor size ≤3 cm, while a limited number of patients (127/716, 17.3%; Table 2) had a tumor larger than 3 cm, though some patients’ MSPLC tumor sizes were unknown (204/716, 28.5%). This implied that most of the patients with MSPLC could be categorized as “early-stage” NSCLC if their tumors were recorded as initial lung cancer, which is a strong indication for sublobar resection and radiation therapy. There were actually only a few patients that underwent lobectomy for MSPLC (entire sample: 352/2451, 14.4%; diagnosed after 2004: 81/716, 11.3%). Radiotherapy seemed to be the most common treatment for MSPLC (entire sample: 864/2451, 35.3%; diagnosed after 2004: 309/716, 43.2%). All the aforementioned facts indicate that most patients with MSPLC might not tolerate another surgical resection, especially lobectomy, or be more willing to receive noninvasive radiation therapy. Therefore, comparing survival outcomes between radiotherapy and wedge resection or lobectomy is highly necessary. As shown in Figure 2, the radiotherapy group also had significantly greater OS than the no treatment group (*P*<.001) but poorer OS than both the lobectomy (*P*=.002) and wedge resection (*P*=.004) groups. When patients’ physical condition and pulmonary function reserve permit, whether choosing lobectomy or wedge resection, patients undergoing surgical resection may gain greater survival benefits than those receiving radiation therapy.

The development of long-term outcome prediction models using ML techniques represents a significant breakthrough in the field of MSPLC. This paper convincingly demonstrates the utility of ML algorithms for accurately predicting cumulative cancer-specific mortality at various time intervals. The exceptional performance of these predictive models emphasizes the superiority of artificial intelligence in prognostic prediction, offering precise and reliable predictions for individual patients. Integrating such models into routine clinical practice has the potential to optimize treatment strategies and improve patient outcomes in MSPLC. Furthermore, the study uses competing risk analysis to delve into the impact of different factors on CSS among MSPLC patients across distinct time intervals. The findings provide valuable insights into the factors influencing both short-term (1-year and 3-year) and long-term (5-year and 10-year) survival outcomes. This enhanced understanding of the factors affecting patient outcomes contributes to improved prognostic assessments and facilitates informed treatment decision-making by clinicians.

Generally, patients with MPLC had better survival outcomes than those with intrapulmonary metastases from IPLC after surgery [22,28]. However, effective methods to accurately identify MPLC patients have not existed until now. Previous studies identified patients with MSPLC using inclusion and exclusion criteria that lacked rigor [8,24,27]. In this study, to avoid the potential confounding effect of metastases, we only included patients with a diagnostic interval greater than 4 years, which indicated a thoroughly representative group of patients with MSPLC [16].

To the best of our knowledge, using PSM analyses and ML techniques on the largest cohort of patients with MSPLC, this study is the first to compare the survival outcomes after radiotherapy with those after surgical resection for MSPLC. Nevertheless, limitations in some aspects of the study still exist. First, this is a retrospective study based on the study population from the SEER database. A certain degree of data bias could not be totally avoided. Second, there might have been an inclination for treatment regarding the patients who received radiotherapy, because they were usually ineligible for surgery due to poorer physical condition and insufficient pulmonary function reserve. Thus, though we tried to control for the confounding effects using PSM, patient bias between different treatment groups also existed because details on physical condition and lung function were unknown. Further evaluation should be performed by prospective studies in the future. Third, since very few patients underwent a pneumonectomy and were thus excluded from our study, the prognostic role of pneumonectomy for patients with MSPLC requires a large cohort to verify. Additionally, we acknowledge the limitations inherent in the SEER database, which lacks comprehensive information, including details on immunotherapy and targeted therapy and the specifics of radiotherapy such as the target volume, treatment dose, and radiation technology. We hope that future cohort studies will incorporate these specifics to provide a more comprehensive understanding of the treatment landscape for MSPLC.

### Conclusions

Overall, this study indicated that surgical resections such as wedge resection and lobectomy performed better than radiation therapy in terms of survival of patients with MSPLC. However, many patients with MSPLC may not tolerate surgery because of previously treated initial lung cancer. Among the treatment options, radiation therapy confers great survival outcomes and can be a valid alternative for surgery. Future prospective studies can be designed to further confirm the effectiveness of radiation therapy for MSPLC.

## Appendix

Multimedia Appendix 1 Supplemental tables.

## Abbreviations

ADB: adaptive boosting
ANN: artificial neural network
AUC of ROCs: area under the receiver operating characteristic curves
CSS: cancer-specific survival
GBDT: gradient boosting decision tree
HR: hazard ratio
ICD-O: International Classification of Diseases for Oncology
IPLC: initial primary lung cancer
KNN: K nearest neighbor
LASSO: least absolute shrinkage and selection operator
ML: machine learning
MPLC: multiple primary lung cancer
MSPLC: metachronous second primary lung cancer
NSCLC: non-small cell lung cancer
OS: overall survival
PSM: propensity score matching
RFC: random forest classifier
SEER: Surveillance, Epidemiology, and End Results
WHO: World Health Organization
XGB: extreme gradient boosting

## Data Availability

The data sets generated and analyzed during the current study are available in the SEER database [14].
