# Supplementary material for: Real-World Survival Comparisons Between Radiotherapy and Surgery for Metachronous Second Primary Lung Cancer and Predictions of Lung Cancer–Specific Outcomes Using Machine Learning: Population-Based Study
Source: JMIR Cancer. 2024 Jun 12;10:e53354. doi: 10.2196/53354 (PMC11208834; doi:10.2196/53354)
Supplement: Multimedia Appendix 1 [file cancer_v10i1e53354_app1.docx]

**Table S1.** Baseline characteristics between radiotherapy and none-treatment for second primary lung cancer before and after propensity score matching (PSM)

| Characteristic | Before PSM | | | After PSM | | |
| --- | --- | --- | --- | --- | --- | --- |
|  | None | Radiation | P value | None | Radiation | P value |
|  | Mean/Count (SD/%) | Mean/Count (SD/%) |  | Mean/Count (SD/%) | Mean/Count (SD/%) |  |
|  | n = 739 | n = 864 |  | n = 563 | n = 563 |  |
| Age, years | 64.8±9.3 | 63.9±8.9 | 0.049 | 64.1±9.3 | 64.4±8.6 | 0.604 |
| Race |  |  |  |  |  |  |
| White | 609 (82.4) | 737 (85.3) | 0.253 | 464 (82.4) | 474 (84.2) | 0.725 |
| Black | 83 (11.2) | 85 (9.8) |  | 65 (11.5) | 58 (10.3) |  |
| Others | 47 (6.4) | 42 (4.9) |  | 34 (6) | 31 (5.5) |  |
| Sex |  |  |  |  |  |  |
| Male | 364 (49.3) | 417 (48.3) | 0.729 | 271 (48.1) | 267 (47.4) | 0.858 |
| Female | 375 (50.7) | 447 (51.7) |  | 292 (51.9) | 296 (52.6) |  |
| Relative location |  |  |  |  |  |  |
| Ipsilateral | 256 (34.6) | 321 (37.2) | 0.321 | 202 (35.9) | 210 (37.3) | 0.665 |
| Contralateral | 483 (65.4) | 543 (62.8) |  | 361 (64.1) | 353 (62.7) |  |
| Diagnosis interval, months | 104.5±48.8) | 104.4 ±48.7 | 0.971 | 106.0±50.4 | 106.6±47.9 | 0.836 |
| **IPLC** |  |  |  |  |  |  |
| Year of diagnosis |  |  |  |  |  |  |
| 1988-1995 | 224 (30.3) | 242 (28) | 0.069 | 174 (30.9) | 166 (29.5) | 0.69 |
| 1996-2003 | 291 (39.4) | 313 (36.2) |  | 210 (37.3) | 224 (39.8) |  |
| 2004-2012 | 224 (30.3) | 309 (35.8) |  | 179 (31.8) | 173 (30.7) |  |
| SEER stage |  |  |  |  |  |  |
| Localized | 411 (55.6) | 560 (64.8) | <.001 | 334 (59.3) | 339 (60.2) | 0.808 |
| Regional | 328 (44.4) | 304 (35.2) |  | 229 (40.7) | 224 (39.8) |  |
| Histology |  |  |  |  |  |  |
| ADC | 405 (54.8) | 451 (52.2) | 0.217 | 308 (54.7) | 309 (54.9) | 0.951 |
| SCC | 210 (28.4) | 280 (32.4) |  | 164 (29.1) | 160 (28.4) |  |
| Other NSCLC | 124 (16.8) | 133 (15.4) |  | 91 (16.2) | 94 (16.7) |  |
| Grade |  |  |  |  |  |  |
| Well differentiated | 79 (10.7) | 82 (9.5) | 0.897 | 56 (9.9) | 53 (9.4) | 0.986 |
| Moderately differentiated | 254 (34.4) | 295 (34.1) |  | 200 (35.5) | 196 (34.8) |  |
| Poorly differentiated | 238 (32.2) | 295 (34.1) |  | 183 (32.5) | 190 (33.7) |  |
| Undifferentiated | 36 (4.9) | 42 (4.9) |  | 27 (4.8) | 25 (4.4) |  |
| Unknown | 132 (17.9) | 150 (17.4) |  | 97 (17.2) | 99 (17.6) |  |
| Surgery |  |  |  |  |  |  |
| No surgery | 111 (15) | 102 (11.8) | 0.234 | 72 (12.8) | 77 (13.7) | 0.948 |
| Sublevel resection | 77 (10.4) | 100 (11.6) |  | 56 (9.9) | 59 (10.5) |  |
| lobectomy | 507 (68.6) | 616 (71.3) |  | 405 (71.9) | 399 (70.9) |  |
| Pneumonectomy | 44 (6) | 46 (5.3) |  | 30 (5.3) | 28 (5) |  |
| Chemotherapy |  |  |  |  |  |  |
| Yes | 164 (22.2) | 155 (17.9) | 0.039 | 110 (19.5) | 109 (19.4) | 1.000 |
| No/unknown | 575 (77.8) | 709 (82.1) |  | 453 (80.5) | 454 (80.6) |  |
| Radiotherapy |  |  |  |  |  |  |
| Yes | 179 (24.2) | 176 (20.4) | 0.073 | 125 (22.2) | 122 (21.7) | 0.885 |
| No/unknown | 560 (75.8) | 688 (79.6) |  | 438 (77.8) | 441 (78.3) |  |
| **SPLC** |  |  |  |  |  |  |
| Chemotherapy |  |  |  |  |  |  |
| Yes | 246 (33.3) | 318 (36.8) | 0.156 | 187 (33.2) | 217 (38.5) | 0.072 |
| No/Unknown | 493 (66.7) | 546 (63.2) |  | 376 (66.8) | 346 (61.5) |  |

^a^IPLC, initial primary lung cancer; SPLC, second primary lung cancer; SD, standard deviation; ADC, adenocarcinoma; SCC, squamous cell carcinoma; NSCLC, non-small cell lung cancer.

**Table S2.** Baseline characteristics between surgery and surgery plus radiotherapy before and after propensity score matching (PSM)

| Characteristic | Before PSM | | | After PSM | | |
| --- | --- | --- | --- | --- | --- | --- |
|  | Radiation + Surgery | Surgery | P value | Radiation + Surgery | Surgery | P value |
|  | Mean/Count (SD/%) | Mean/Count (SD/%) |  | Mean/Count (SD/%) | Mean/Count (SD/%) |  |
|  | n = 89 | n = 759 |  | n = 69 | n = 69 |  |
| Age, years | 60.6 (9.3) | 62.1 (9.0) | 0.152 | 61.4 (9.0) | 61.2 (8.4) | 0.899 |
| Race |  |  |  |  |  |  |
| White | 74 (83.1) | 642 (84.6) | 0.842 | 58 (84.1) | 62 (89.9) | 0.537 |
| Black | 9 (10.1) | 63 (8.3) |  | 5 (7.2) | 4 (5.8) |  |
| Others | 6 (6.7) | 54 (7.1) |  | 6 (8.7) | 3 (4.3) |  |
| Sex |  |  |  |  |  |  |
| Male | 43 (48.3) | 313 (41.2) | 0.244 | 32 (46.4) | 38 (55.1) | 0.395 |
| Female | 46 (51.7) | 446 (58.8) |  | 37 (53.6) | 31 (44.9) |  |
| Relative location |  |  |  |  |  |  |
| Ipsilateral | 30 (33.7) | 208 (27.4) | 0.26 | 21 (30.4) | 19 (27.5) | 0.851 |
| Contralateral | 59 (66.3) | 551 (72.6) |  | 48 (69.6) | 50 (72.5) |  |
| Diagnosis interval, months | 82.9 (35.9) | 95.8 (45.3) | 0.01 | 85.1 (37.1) | 87.3 (37.9) | 0.734 |
| **IPLC** |  |  |  |  |  |  |
| Year of diagnosis |  |  |  |  |  |  |
| 1988-1995 | 41 (46.1) | 256 (33.7) | 0.063 | 27 (39.1) | 29 (42) | 0.926 |
| 1996-2003 | 29 (32.6) | 286 (37.7) |  | 25 (36.2) | 23 (33.3) |  |
| 2004-2012 | 19 (21.3) | 217 (28.6) |  | 17 (24.6) | 17 (24.6) |  |
| SEER stage |  |  |  |  |  |  |
| Localized | 62 (69.7) | 505 (66.5) | 0.635 | 46 (66.7) | 43 (62.3) | 0.722 |
| Regional | 27 (30.3) | 254 (33.5) |  | 23 (33.3) | 26 (37.7) |  |
| Histology |  |  |  |  |  |  |
| ADC | 48 (53.9) | 495 (65.2) | 0.11 | 38 (55.1) | 37 (53.6) | 0.968 |
| SCC | 27 (30.3) | 173 (22.8) |  | 22 (31.9) | 22 (31.9) |  |
| Other NSCLC | 14 (15.7) | 91 (12) |  | 9 (13) | 10 (14.5) |  |
| Grade |  |  |  |  |  |  |
| Well differentiated | 10 (11.2) | 106 (14) | 0.65 | 6 (8.7) | 8 (11.6) | 0.488 |
| Moderately differentiated | 36 (40.4) | 259 (34.1) |  | 28 (40.6) | 34 (49.3) |  |
| Poorly differentiated | 28 (31.5) | 231 (30.4) |  | 23 (33.3) | 17 (24.6) |  |
| Undifferentiated | 4 (4.5) | 33 (4.3) |  | 2 (2.9) | 4 (5.8) |  |
| Unknown | 11 (12.4) | 130 (17.1) |  | 10 (14.5) | 6 (8.7) |  |
| Surgery |  |  |  |  |  |  |
| No surgery | 4 (4.5) | 47 (6.2) | 0.496 | 3 (4.3) | 5 (7.2) | 0.655 |
| Sublevel resection | 13 (14.6) | 105 (13.8) |  | 9 (13) | 11 (15.9) |  |
| lobectomy | 72 (80.9) | 591 (77.9) |  | 57 (82.6) | 53 (76.8) |  |
| Pneumonectomy | 0 (0) | 16 (2.1) |  | 0 (0) | 0 (0) |  |
| Chemotherapy |  |  |  |  |  |  |
| Yes | 15 (16.9) | 131 (17.3) | 1.000 | 12 (17.4) | 18 (26.1) | 0.302 |
| No/unknown | 74 (83.1) | 628 (82.7) |  | 57 (82.6) | 51 (73.9) |  |
| Radiotherapy |  |  |  |  |  |  |
| Yes | 11 (12.4) | 123 (16.2) | 0.431 | 8 (11.6) | 13 (18.8) | 0.343 |
| No/unknown | 78 (87.6) | 636 (83.8) |  | 61 (88.4) | 56 (81.2) |  |
| **SPLC** |  |  |  |  |  |  |
| Chemotherapy |  |  |  |  |  |  |
| Yes | 39 (43.8) | 91 (12) | <.001 | 26 (37.7) | 15 (21.7) | 0.062 |
| No/Unknown | 50 (56.2) | 668 (88) |  | 43 (62.3) | 54 (78.3) |  |

^a^IPLC, initial primary lung cancer; SPLC, second primary lung cancer; SD, standard deviation; ADC, adenocarcinoma; SCC, squamous cell carcinoma; NSCLC, non-small cell lung cancer.

**Table S3.** Baseline characteristics between wedge resection and radiotherapy for second primary lung cancer before and after propensity score matching (PSM) after 2004

| Characteristic | Before PSM | | | After PSM | | |
| --- | --- | --- | --- | --- | --- | --- |
|  | Wedge | Radiation | P value | Wedge | Radiation | P value |
|  | Mean/Count (SD/%) | Mean/Count (SD/%) |  | Mean/Count (SD/%) | Mean/Count (SD/%) |  |
|  | n = 102 | n = 309 |  | n = 85 | n = 85 |  |
| Age, years | 64.3±8.8 | 66.4±8.9 | 0.037 | 65.0±8.7 | 65.2±8.4 | 0.907 |
| Race |  |  |  |  |  |  |
| White | 94 (92.2) | 269 (87.1) | 0.212 | 77 (90.6) | 78 (91.8) | 0.928 |
| Black | 4 (3.9) | 29 (9.4) |  | 4 (4.7) | 4 (4.7) |  |
| Others | 4 (3.9) | 11 (3.6) |  | 4 (4.7) | 3 (3.5) |  |
| Sex |  |  |  |  |  |  |
| Male | 27 (26.5) | 131 (42.4) | 0.006 | 26 (30.6) | 22 (25.9) | 0.609 |
| Female | 75 (73.5) | 178 (57.6) |  | 59 (69.4) | 63 (74.1) |  |
| Relative location |  |  |  |  |  |  |
| Ipsilateral | 31 (30.4) | 126 (40.8) | 0.079 | 27 (31.8) | 21 (24.7) | 0.394 |
| Contralateral | 71 (69.6) | 183 (59.2) |  | 58 (68.2) | 64 (75.3) |  |
| Diagnosis interval, months | 71.2±18.8 | 75.7±22.2 | 0.064 | 71.9±18.8 | 72.4±21.2 | 0.872 |
| **IPLC** |  |  |  |  |  |  |
| T stage |  |  |  |  |  |  |
| T1 | 44 (43.1) | 142 (46) | 0.967 | 35 (41.2) | 32 (37.6) | 0.906 |
| T2 | 41 (40.2) | 114 (36.9) |  | 33 (38.8) | 38 (44.7) |  |
| T3 | 5 (4.9) | 14 (4.5) |  | 5 (5.9) | 6 (7.1) |  |
| T4 | 9 (8.8) | 27 (8.7) |  | 9 (10.6) | 7 (8.2) |  |
| Unknown | 3 (2.9) | 12 (3.9) |  | 3 (3.5) | 2 (2.4) |  |
| N stage |  |  |  |  |  |  |
| N0 | 79 (77.5) | 238 (77) | 0.941 | 64 (75.3) | 69 (81.2) | 0.55 |
| N1 | 10 (9.8) | 29 (9.4) |  | 9 (10.6) | 4 (4.7) |  |
| N2 | 11 (10.8) | 38 (12.3) |  | 10 (11.8) | 10 (11.8) |  |
| Unknown | 2 (2) | 4 (1.3) |  | 2 (2.4) | 2 (2.4) |  |
| Histology |  |  |  |  |  |  |
| ADC | 69 (67.6) | 165 (53.4) | 0.023 | 54 (63.5) | 48 (56.5) | 0.64 |
| SCC | 19 (18.6) | 99 (32) |  | 18 (21.2) | 21 (24.7) |  |
| Other NSCLC | 14 (13.7) | 45 (14.6) |  | 13 (15.3) | 16 (18.8) |  |
| Grade |  |  |  |  |  |  |
| Well differentiated | 16 (15.7) | 41 (13.3) | 0.763 | 13 (15.3) | 12 (14.1) | 0.895 |
| Moderately differentiated | 35 (34.3) | 117 (37.9) |  | 31 (36.5) | 29 (34.1) |  |
| Poorly differentiated | 32 (31.4) | 90 (29.1) |  | 25 (29.4) | 30 (35.3) |  |
| Undifferentiated | 4 (3.9) | 7 (2.3) |  | 3 (3.5) | 4 (4.7) |  |
| Unknown | 15 (14.7) | 54 (17.5) |  | 13 (15.3) | 10 (11.8) |  |
| Surgery |  |  |  |  |  |  |
| No surgery | 6 (5.9) | 59 (19.1) | 0.011 | 6 (7.1) | 6 (7.1) | 0.952 |
| Sublevel resection | 15 (14.7) | 47 (15.2) |  | 13 (15.3) | 13 (15.3) |  |
| lobectomy | 79 (77.5) | 194 (62.8) |  | 64 (75.3) | 65 (76.5) |  |
| Pneumonectomy | 2 (2.0) | 9 (2.9) |  | 2 (2.4) | 1 (1.2) |  |
| Chemotherapy |  |  |  |  |  |  |
| Yes | 32 (31.4) | 97 (31.4) | 1.000 | 26 (30.6) | 30 (35.3) | 0.624 |
| No/unknown | 70 (68.6) | 212 (68.6) |  | 59 (69.4) | 55 (64.7) |  |
| Radiotherapy |  |  |  |  |  |  |
| Yes | 15 (14.7) | 87 (28.2) | 0.009 | 13 (15.3) | 16 (18.8) | 0.683 |
| No/unknown | 87 (85.3) | 222 (71.8) |  | 72 (84.7) | 69 (81.2) |  |
| **SPLC** |  |  |  |  |  |  |
| Tumor size |  |  |  |  |  |  |
| < 3 cm | 85 (83.3) | 163 (52.8) | <.001 | 69 (81.2) | 68 (80) | 0.913 |
| 3-5 cm | 4 (3.9) | 33 (10.7) |  | 4 (4.7) | 3 (3.5) |  |
| > 5 cm | 1 (1.0) | 22 (7.1) |  | 1 (1.2) | 2 (2.4) |  |
| Unknown | 12 (11.8) | 91 (29.4) |  | 11 (12.9) | 12 (14.1) |  |
| Chemotherapy |  |  |  |  |  |  |
| Yes | 15 (14.7) | 79 (25.6) | 0.033 | 13 (15.3) | 19 (22.4) | 0.327 |
| No/Unknown | 87 (85.3) | 230 (74.4) |  | 72 (84.7) | 66 (77.6) |  |

^a^IPLC, initial primary lung cancer; SPLC, second primary lung cancer; SD, standard deviation; ADC, adenocarcinoma; SCC, squamous cell carcinoma; NSCLC, non-small cell lung cancer.

**Table S4.** Baseline characteristics between lobectomy and radiotherapy for second primary lung cancer before and after propensity score matching (PSM) after 2004

| Characteristic | Before PSM | | | After PSM | | |
| --- | --- | --- | --- | --- | --- | --- |
|  | Lobectomy | Radiation | P value | Lobectomy | Radiation | P value |
|  | Mean/Count (SD/%) | Mean/Count (SD/%) |  | Mean/Count (SD/%) | Mean/Count (SD/%) |  |
|  | n = 81 | n = 309 |  | n = 71 | n = 71 |  |
| Age, years | 62.9 (7.6) | 66.4 (8.9) | 0.001 | 63.9 (7.0) | 65.0 (8.1) | 0.388 |
| Race |  |  |  |  |  |  |
| White | 65 (80.2) | 269 (87.1) | 0.023 | 59 (83.1) | 62 (87.3) | 0.584 |
| Black | 7 (8.6) | 29 (9.4) |  | 6 (8.5) | 6 (8.5) |  |
| Others | 9 (11.1) | 11 (3.6) |  | 6 (8.5) | 3 (4.2) |  |
| Sex |  |  |  |  |  |  |
| Male | 38 (46.9) | 131 (42.4) | 0.545 | 33 (46.5) | 31 (43.7) | 0.866 |
| Female | 43 (53.1) | 178 (57.6) |  | 38 (53.5) | 40 (56.3) |  |
| Relative location |  |  |  |  |  |  |
| Ipsilateral | 28 (34.6) | 126 (40.8) | 0.374 | 23 (32.4) | 25 (35.2) | 0.859 |
| Contralateral | 53 (65.4) | 183 (59.2) |  | 48 (67.6) | 46 (64.8) |  |
| Diagnosis interval, months | 70.2 (21.8) | 75.7 (22.2) | 0.044 | 71.1 (20.8) | 66.9 (19.4) | 0.209 |
| **IPLC** |  |  |  |  |  |  |
| T stage |  |  |  |  |  |  |
| T1 | 41 (50.6) | 142 (46) | 0.21 | 35 (49.3) | 39 (54.9) | 0.75 |
| T2 | 30 (37) | 114 (36.9) |  | 28 (39.4) | 24 (33.8) |  |
| T3 | 5 (6.2) | 14 (4.5) |  | 4 (5.6) | 6 (8.5) |  |
| T4 | 1 (1.2) | 27 (8.7) |  | 1 (1.4) | 1 (1.4) |  |
| Unknown | 4 (4.9) | 12 (3.9) |  | 3 (4.2) | 1 (1.4) |  |
| N stage |  |  |  |  |  |  |
| N0 | 63 (77.8) | 238 (77) | 0.417 | 54 (76.1) | 55 (77.5) | 0.922 |
| N1 | 5 (6.2) | 29 (9.4) |  | 5 (7) | 6 (8.5) |  |
| N2 | 10 (12.3) | 38 (12.3) |  | 10 (14.1) | 9 (12.7) |  |
| Unknown | 3 (3.7) | 4 (1.3) |  | 2 (2.8) | 1 (1.4) |  |
| Histology |  |  |  |  |  |  |
| ADC | 46 (56.8) | 165 (53.4) | 0.862 | 38 (53.5) | 42 (59.2) | 0.748 |
| SCC | 24 (29.6) | 99 (32) |  | 23 (32.4) | 19 (26.8) |  |
| Other NSCLC | 11 (13.6) | 45 (14.6) |  | 10 (14.1) | 10 (14.1) |  |
| Grade |  |  |  |  |  |  |
| Well differentiated | 8 (9.9) | 41 (13.3) | 0.584 | 8 (11.3) | 8 (11.3) | 0.987 |
| Moderately differentiated | 32 (39.5) | 117 (37.9) |  | 26 (36.6) | 25 (35.2) |  |
| Poorly differentiated | 27 (33.3) | 90 (29.1) |  | 24 (33.8) | 26 (36.6) |  |
| Undifferentiated | 0 (0) | 7 (2.3) |  | 0 (0) | 0 (0) |  |
| Unknown | 14 (17.3) | 54 (17.5) |  | 13 (18.3) | 12 (16.9) |  |
| Surgery |  |  |  |  |  |  |
| No surgery | 7 (8.6) | 59 (19.1) | 0.028 | 7 (9.9) | 8 (11.3) | 0.963 |
| Sublevel resection | 21 (25.9) | 47 (15.2) |  | 16 (22.5) | 18 (25.4) |  |
| lobectomy | 52 (64.2) | 194 (62.8) |  | 47 (66.2) | 44 (62) |  |
| Pneumonectomy | 1 (1.2) | 9 (2.9) |  | 1 (1.4) | 1 (1.4) |  |
| Chemotherapy |  |  |  |  |  |  |
| Yes | 24 (29.6) | 97 (31.4) | 0.865 | 19 (26.8) | 19 (26.8) | 1.000 |
| No/unknown | 57 (70.4) | 212 (68.6) |  | 52 (73.2) | 52 (73.2) |  |
| Radiotherapy |  |  |  |  |  |  |
| Yes | 12 (14.8) | 87 (28.2) | 0.021 | 12 (16.9) | 8 (11.3) | 0.469 |
| No/unknown | 69 (85.2) | 222 (71.8) |  | 59 (83.1) | 63 (88.7) |  |
| **SPLC** |  |  |  |  |  |  |
| Tumor size |  |  |  |  |  |  |
| 0-3 cm | 51 (63) | 163 (52.8) | 0.030 | 45 (63.4) | 43 (60.6) | 0.931 |
| 3-5 cm | 13 (16) | 33 (10.7) |  | 11 (15.5) | 11 (15.5) |  |
| > 5 cm | 6 (7.4) | 22 (7.1) |  | 4 (5.6) | 6 (8.5) |  |
| Unknown | 11 (13.6) | 91 (29.4) |  | 11 (15.5) | 11 (15.5) |  |
| Chemotherapy |  |  |  |  |  |  |
| Yes | 21 (25.9) | 79 (25.6) | 1.000 | 13 (18.3) | 20 (28.2) | 0.233 |
| No/Unknown | 60 (74.1) | 230 (74.4) |  | 58 (81.7) | 51 (71.8) |  |

^a^IPLC, initial primary lung cancer; SPLC, second primary lung cancer; SD, standard deviation; ADC, adenocarcinoma; SCC, squamous cell carcinoma; NSCLC, non-small cell lung cancer.

**Table S5.** Baseline characteristics between radiotherapy and none-treatment for second primary lung cancer before and after propensity score matching (PSM) after 2004

| Characteristic | Before PSM | | | After PSM | | |
| --- | --- | --- | --- | --- | --- | --- |
|  | Lobectomy | Radiation | P value | Lobectomy | Radiation | P value |
|  | Mean/Count (SD/%) | Mean/Count (SD/%) |  | Mean/Count (SD/%) | Mean/Count (SD/%) |  |
|  | n = 81 | n = 309 |  | n = 71 | n = 71 |  |
| Age, years | 62.9 (7.6) | 66.4 (8.9) | 0.001 | 63.9 (7.0) | 65.0 (8.1) | 0.388 |
| Race |  |  |  |  |  |  |
| White | 65 (80.2) | 269 (87.1) | 0.023 | 59 (83.1) | 62 (87.3) | 0.584 |
| Black | 7 (8.6) | 29 (9.4) |  | 6 (8.5) | 6 (8.5) |  |
| Others | 9 (11.1) | 11 (3.6) |  | 6 (8.5) | 3 (4.2) |  |
| Sex |  |  |  |  |  |  |
| Male | 38 (46.9) | 131 (42.4) | 0.545 | 33 (46.5) | 31 (43.7) | 0.866 |
| Female | 43 (53.1) | 178 (57.6) |  | 38 (53.5) | 40 (56.3) |  |
| Relative location |  |  |  |  |  |  |
| Ipsilateral | 28 (34.6) | 126 (40.8) | 0.374 | 23 (32.4) | 25 (35.2) | 0.859 |
| Contralateral | 53 (65.4) | 183 (59.2) |  | 48 (67.6) | 46 (64.8) |  |
| Diagnosis interval, months | 70.2 (21.8) | 75.7 (22.2) | 0.044 | 71.1 (20.8) | 66.9 (19.4) | 0.209 |
| **IPLC** |  |  |  |  |  |  |
| T stage |  |  |  |  |  |  |
| T1 | 41 (50.6) | 142 (46) | 0.21 | 35 (49.3) | 39 (54.9) | 0.75 |
| T2 | 30 (37) | 114 (36.9) |  | 28 (39.4) | 24 (33.8) |  |
| T3 | 5 (6.2) | 14 (4.5) |  | 4 (5.6) | 6 (8.5) |  |
| T4 | 1 (1.2) | 27 (8.7) |  | 1 (1.4) | 1 (1.4) |  |
| Unknown | 4 (4.9) | 12 (3.9) |  | 3 (4.2) | 1 (1.4) |  |
| N stage |  |  |  |  |  |  |
| N0 | 63 (77.8) | 238 (77) | 0.417 | 54 (76.1) | 55 (77.5) | 0.922 |
| N1 | 5 (6.2) | 29 (9.4) |  | 5 (7) | 6 (8.5) |  |
| N2 | 10 (12.3) | 38 (12.3) |  | 10 (14.1) | 9 (12.7) |  |
| Unknown | 3 (3.7) | 4 (1.3) |  | 2 (2.8) | 1 (1.4) |  |
| Histology |  |  |  |  |  |  |
| ADC | 46 (56.8) | 165 (53.4) | 0.862 | 38 (53.5) | 42 (59.2) | 0.748 |
| SCC | 24 (29.6) | 99 (32) |  | 23 (32.4) | 19 (26.8) |  |
| Other NSCLC | 11 (13.6) | 45 (14.6) |  | 10 (14.1) | 10 (14.1) |  |
| Grade |  |  |  |  |  |  |
| Well differentiated | 8 (9.9) | 41 (13.3) | 0.584 | 8 (11.3) | 8 (11.3) | 0.987 |
| Moderately differentiated | 32 (39.5) | 117 (37.9) |  | 26 (36.6) | 25 (35.2) |  |
| Poorly differentiated | 27 (33.3) | 90 (29.1) |  | 24 (33.8) | 26 (36.6) |  |
| Undifferentiated | 0 (0) | 7 (2.3) |  | 0 (0) | 0 (0) |  |
| Unknown | 14 (17.3) | 54 (17.5) |  | 13 (18.3) | 12 (16.9) |  |
| Surgery |  |  |  |  |  |  |
| No surgery | 7 (8.6) | 59 (19.1) | 0.028 | 7 (9.9) | 8 (11.3) | 0.963 |
| Sublevel resection | 21 (25.9) | 47 (15.2) |  | 16 (22.5) | 18 (25.4) |  |
| lobectomy | 52 (64.2) | 194 (62.8) |  | 47 (66.2) | 44 (62) |  |
| Pneumonectomy | 1 (1.2) | 9 (2.9) |  | 1 (1.4) | 1 (1.4) |  |
| Chemotherapy |  |  |  |  |  |  |
| Yes | 24 (29.6) | 97 (31.4) | 0.865 | 19 (26.8) | 19 (26.8) | 1.000 |
| No/unknown | 57 (70.4) | 212 (68.6) |  | 52 (73.2) | 52 (73.2) |  |
| Radiotherapy |  |  |  |  |  |  |
| Yes | 12 (14.8) | 87 (28.2) | 0.021 | 12 (16.9) | 8 (11.3) | 0.469 |
| No/unknown | 69 (85.2) | 222 (71.8) |  | 59 (83.1) | 63 (88.7) |  |
| **SPLC** |  |  |  |  |  |  |
| Tumor size |  |  |  |  |  |  |
| 0-3 cm | 51 (63) | 163 (52.8) | 0.030 | 45 (63.4) | 43 (60.6) | 0.931 |
| 3-5 cm | 13 (16) | 33 (10.7) |  | 11 (15.5) | 11 (15.5) |  |
| > 5 cm | 6 (7.4) | 22 (7.1) |  | 4 (5.6) | 6 (8.5) |  |
| Unknown | 11 (13.6) | 91 (29.4) |  | 11 (15.5) | 11 (15.5) |  |
| Chemotherapy |  |  |  |  |  |  |
| Yes | 21 (25.9) | 79 (25.6) | 1.000 | 13 (18.3) | 20 (28.2) | 0.233 |
| No/Unknown | 60 (74.1) | 230 (74.4) |  | 58 (81.7) | 51 (71.8) |  |

^a^IPLC, initial primary lung cancer; SPLC, second primary lung cancer; SD, standard deviation; ADC, adenocarcinoma; SCC, squamous cell carcinoma; NSCLC, non-small cell lung cancer.
